# The complete chloroplast genome sequence of *Dendrocalamus sinicus*

**DOI:** 10.1080/23802359.2019.1664947

**Published:** 2019-09-12

**Authors:** Yi Wang, Xiaolong Yuan, Yunqing Li, Jinfeng Zhang

**Affiliations:** Laboratory of Forest Plant Cultivation and Utilization, Yunnan Academy of Forestry, Kunming, Yunnan, People's Republic of China

**Keywords:** *Dendrocalamus sinicus*, chloroplast, Illumina sequencing, phylogenetic analysis

## Abstract

*Dendrocalamus sinicus* is the world’s largest bamboo species. In this study, the complete chloroplast genome (cpDNA) sequence of *D. sinicus* was determined from Illumina HiSeq pair-end sequencing data. The cpDNA is 139,441 bp in length, contains a large single-copy region (LSC) of 82,777 bp and a small single-copy region (SSC) of 12,803 bp, which were separated by a pair of inverted repeat (IR) regions of 21,296 bp. The genome contains 136 genes, including 89 protein-coding genes, 8 ribosomal RNA genes, and 40 transfer RNA genes. The overall GC content of the whole genome is 38.9%, and the corresponding values of the LSC, SSC, and IR regions are 37%, 33.2%, and 44.2%, respectively. Further, phylogenomic analysis showed that *D. sinicus* clustered together with *D. latiflorus*.

*Dendrocalamus sinicus* is the species of the genus Dendrocalamus within the family Gramineae and subfamily Bambusoideae (Geng et al. 2006). *Dendrocalamus sinicus* is the world’s largest bamboo species with strong woody culms (Cui et al. 2016), It can grow to more than 30 m in height and 30 cm in diameter in a short time (Dong et al. 2012). *Dendrocalamus sinicus* can grow quickly and has high productivity, it also is considered as one of the most potential renewable non-woody lignocellulosic feedstocks for bioenergy and biorefinery (Shi et al. 2013). However, there has been no genomic studies on *D. sinicus*.

Herein, we reported and characterized the complete *D. sinicus* plastid genome (MK962316). One *D. sinicus* individual (specimen number: 2015060171) was collected from Puer, Yunnan Province of China (23°78′15″ N, 101°38′19″ E). The specimen is stored at Yunnan Academy of Forestry Herbarium, Kunming, China, and the accession number is YAFH0012413. DNA was extracted from its fresh leaves using DNA Plantzol Reagent (Invitrogen, Carlsbad, CA, USA).

Paired-end reads were sequenced by using Illumina HiSeq system (Illumina, San Diego, CA). In total, about 32.6 million high-quality clean reads were generated with adaptors trimmed. Aligning, assembly, and annotation were conducted by CLC de novo assembler (CLC Bio, Aarhus, Denmark), BLAST, GeSeq (Tillich et al. 2017), and GENEIOUS v 11.0.5 (Biomatters Ltd, Auckland, New Zealand). To confirm the phylogenetic position of *D. sinicus*, other eighteen species of genus Bambuseae from NCBI were aligned using MAFFT v.7 (Katoh and Standley 2013) and maximum likelihood (ML) bootstrap analysis was conducted using RAxML (Stamatakis 2006); bootstrap probability values were calculated from 1000 replicates. *Buergersiochloa bambusoides* (KJ871000) was served as the out-group.

The complete *D. sinicus* plastid genome is a circular DNA molecule with the length of 139,441 bp, contains a large single-copy region (LSC) of 82,777 bp and a small single-copy region (SSC) of 12,803 bp, which were separated by a pair of inverted repeat (IR) regions of 21,296 bp. The overall GC content of the whole genome is 38.9%, and the corresponding values of the LSC, SSC, and IR regions are 37%, 33.2%, and 44.2%, respectively. The plastid genome contained 136 genes, including 89 protein-coding genes, 8 ribosomal RNA genes, and 40 transfer RNA genes. Phylogenetic analysis showed that *D. sinicus* clustered together with *D. latiflorus* (Figure 1). The determination of the complete plastid genome sequences provided new molecular data to illuminate the *Bambuseae* evolution.

**Figure 1.**
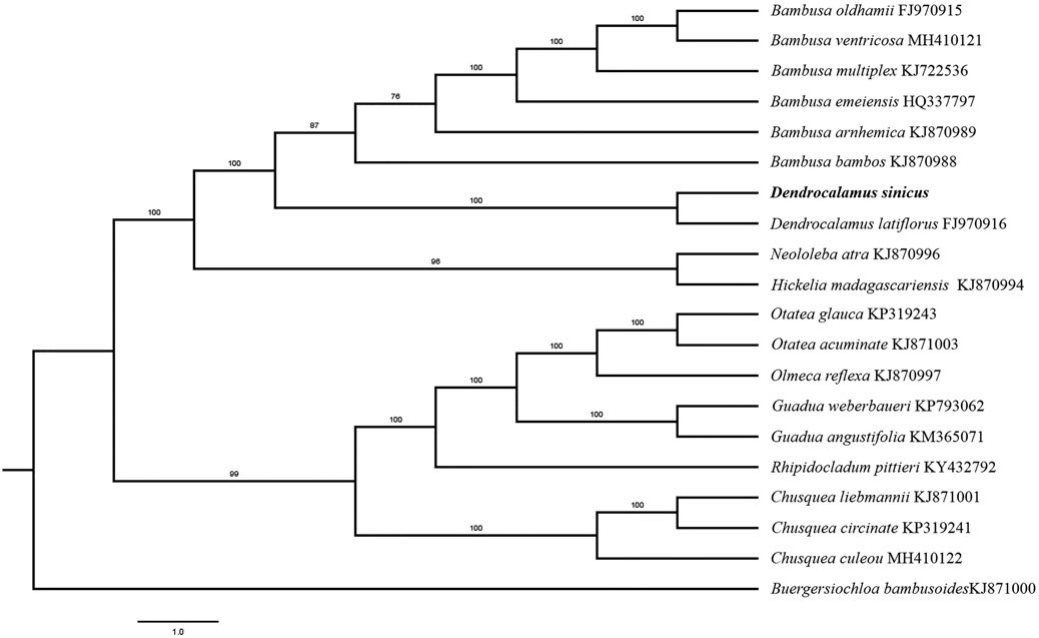
The maximum-likelihood tree based on the 19 chloroplast genomes of *Bambuseae*. The bootstrap value based on 1000 replicates is shown on each node.
